# Multiple lines of evidence reveal a new species of Krait (Squamata, Elapidae, *Bungarus*) from Southwestern China and Northern Myanmar

**DOI:** 10.3897/zookeys.1025.62305

**Published:** 2021-03-18

**Authors:** Ze-Ning Chen, Sheng-Chao Shi, Gernot Vogel, Li Ding, Jing-Song Shi

**Affiliations:** 1 Chengdu Institute of Biology, Chinese Academy of Sciences, Chengdu, Sichuan 610041, China Chengdu Institute of Biology, Chinese Academy of Sciences Chengdu China; 2 Guangxi Key Laboratory of Rare and Endangered Animal Ecology, Guangxi Normal University, Guilin, Guangxi 541001, China Guangxi Normal University Guilin China; 3 University of Chinese Academy of Sciences, Beijing 100049, China University of Chinese Academy of Sciences Beijing China; 4 Society for Southeast Asian Herpetology, Im Sand 3, Heidelberg D-69115, Germany Society for Southeast Asian Herpetology Heidelberg Germany; 5 Key Laboratory of Vertebrate Evolution and Human Origins of Chinese Academy of Sciences, Institute of Vertebrate Paleontology and Paleoanthropology, Chinese Academy of Sciences, Beijing 100044, China Origins of Chinese Academy of Sciences Beijing China

**Keywords:** *Bungarus suzhenae* sp. nov., cranial osteology, hemipenial morphology, micro-computed tomography, phylogeny, taxonomy

## Abstract

Kraits of the genus *Bungarus* Daudin 1803 are widely known venomous snakes distributed from Iran to China and Indonesia. Here, we use a combination of mitochondrial DNA sequence data and morphological data to describe a new species from Yingjiang County, Yunnan Province, China: *Bungarus suzhenae***sp. nov**. Phylogenetically, this species forms a monophyletic lineage sister to the *Bungarus candidus*/*multicinctus*/*wanghaotingi* complex based on cyt *b* and ND4 genes but forms a sister species pair with the species *B. magnimaculatus* Wall & Evans, 1901 based on COI gene fragments. Morphologically, *B. suzhenae***sp. nov**. is similar to the *B. candidus*/*multicinctus*/*wanghaotingi* complex but differs from these taxa by a combination of dental morphology, squamation, coloration pattern, as well as hemipenial morphology. A detailed description of the cranial osteology of the new species is given based on micro-CT tomography images. We revised the morphological characters of *B. candidus*/*multicinctus*/*wanghaotingi* complex and verified the validity of three species in this complex. The distribution of these species was revised; the records of *B. candidus* in China should be attributed to *B. wanghaotingi*. We also provide an updated key to species of *Bungarus*.

## Introduction

Sound taxonomy of lethal snakes provides an essential foundation for venom research, antivenin development and proper snakebite treatment (Fry et al. 2003; Williams et al. 2011). *Bungarus* Daudin 1803 (commonly referred to as ‘Kraits’) is one of the most medically significant groups of elapid snakes in Asia, and is widely distributed from Iran and Pakistan, eastwards to China and Indonesia (Slowinski 1994; Kuch et al. 2005; Abtin et al. 2014; Ahsan and Rahman 2017; Uetz et al. 2020). Within the fourteen species currently described, *Bungarus* with black-and-white crossbands have been the most taxonomically problematic members of the genus and are difficult to identify in the field due to overlapping characteristics in morphology (Wall 1907; Slowinski 1994; Kuch 2007; Abtin et al. 2014). Most of these black-and-white *Bungarus* species are traditionally identified by the number of the dorsal crossbands on the body and tail (Slowinski 1994; Leviton et al. 2003; Zhao 2006). However, both characters within these species overlap (see: Table 1) and may lead to misidentifications.

**Table 1.** Comparison of main morphological characters in the *Bungarus* species.

Species	DSR	VEN	NSC	SC	Loreal	Dorsal body pattern	BB	Reference
***B. candidus***	15	209–224	41–50	undivided	absent	white bands	18–26	This study
		(215.2 ± 3.8 n = 18)	(46.2 ± 2.1 n = 17)				(21.4 ± 1.8 n = 19)	
***B. wanghaotingi***	15	209–259	32–64	undivided	absent	white bands	18–33	This study (n = 16); Yang and Rao 2008 (n = 10); Pope 1928 (n = 1)
		(230.4 ± 12.3 n = 23)	(51.1 ± 6.4 n = 22)				(25.1 ± 3.2 n = 27)	
***B. multicinctus***	15	196–236	38–58	undivided	absent	white bands	31–50	This study
		(214.1 ± 8.9 n = 24)	(47.1 ± 4.9 n = 23)				(39.3 ± 4.7 n = 24)	
***B. suzhenae* sp. nov.**	15	220–229	51–54	undivided	absent	white bands	26–38	This study
		(223 ± 4.1 n = 4)	(53 ± 1.5 n = 3)				(39.3 ± 4.7 n = 4)	
***B. magnimaculatus***	15	214–235 (n = ?)	40–48	undivided	absent	broad, white bands	11–14	Leviton et al. 2003
***B. niger***	15	216–231 (n = ?)	47–57	undivided	absent	body black	/	Wall 1908
***B. caeruleus***	15	200–217 (n > 20)	33–54	undivided	absent	white bands (in pairs)	29–65	Biswas and Sanyal 1978 (n = ?); Slowinski 1994 (n = 11); Whitaker and Captain 2004 (n = ?); This study (n = 9)
***B. ceylonicus***	15	219–235 (n = ?)	33–40	undivided	absent	narrow white rings	15–20	Boulenger 1890; Wall 1908; this study
***B. lividus***	15	212–225 (n = ?)	37–56	undivided	absent	black or blackish blue	/	Smits 1943
***B. andamanensis***	15	193–197 (n = ?)	45–47	undivided	absent	yellow band	39–47	Biswas and Sanyal 1978
***B. persicus***	17	236–238 (n = 2)	50–53	undivided	present	white crossbars	25	Abtin et al. 2014
***B. sindanus***	17	220–237 (n = ?)	45–53	undivided	absent	white bands	/	Khan 2002
***B. walli***	17	196–208 (n = ?)	50–55	undivided	absent	white spots	/	Wall 1907
***B. fasciatus***	15	217–237 (n > 11)	33–41	undivided	absent	yellow band	19–29	Yang and Rao 2008; Leviton et al. 2003; and this study
***B. slowinskii***	15	225–230 (n = 7)	33–41	divided	absent	narrow white rings	27–33	Kuch et al. 2005 (n = 3); Kharin et al. 2011 (n = 3); Smits and Hauser 2019 (n =1)
***B. bungaroides***	15	220–237 (n = ?)	44–51	divided	absent	white rings	46–60	Kuch et al. 2005; Kharin et al. 2011; Smits and Hauser 2019
***B. flaviceps***	13	193–236 (n = ?)	42–52	undivided	absent	head red or orange, body not black and white banded	/	Wall 1908; Kuch 2007

Abbreviations. – See in Material and methods. Note: In the *B. candidus*/*multicinctus* complex, the values in parentheses represent the mean and standard deviation, and some specimens are incomplete; we only count complete specimens for certain characteristics.

Five taxa of *Bungarus* are reported from China, including *B. fasciatus* (Schneider, 1801), *B. bungaroides* (Cantor, 1839), *B. multicinctus multicinctus* Blyth, 1860, *B. m. wanghaotingi* Pope, 1928 and *B. candidus* (Linnaeus, 1758) (Pope 1928; Zhao and Yang 1997; Rao and Zhao 2004; Zhao 2006; Kuch 2007; Yang and Rao 2008; Xie et al. 2018). The former two species *B. fasciatus* and *B. bungaroides* are easily distinguished from their congeners by having divided subcaudal scales, as well as their unique coloration patterns (Kuch et al. 2005; Zhao 2006; Kuch 2007; Yang and Rao 2008). However, *B. m. multicinctus*, *B. m. wanghaotingi* and *B. candidus* are relatively difficult to identify and are differentiated from each other by the number of the white crossbands on the body and the number of ventral scales (Pope 1928; Slowinski 1994; Leviton et al. 2003; Rao and Zhao 2004; Zhao 2006). Leviton et al. (2003) proposed to raise *B. m. wanghaotingi* to species level based on lesser light crossbands on body and tail and geographically distant from *B. m. multicinctus*. However, Kuch (2007) considered *B. candidus* and *B. multicinctus* (including *B. m. multicinctus* and *B. m. wanghaotingi*) as a species complex based on mtDNA sequence data and similar morphology. Considering paraphyly of this complex shown in Kuch (2007), we use the term *B. candidus*/*multicinctus*/*wanghaotingi* complex. In addition, Kuch (2007) tentatively identified a specimen CAS 221526 from northern Myanmar as Bungarus cf. multicinctus, which is phylogenetically closer to *B. niger* Wall, 1908 than to all other specimens of *B. multicinctus* from China and Vietnam (fig. 20 in Kuch 2007). Since Kuch’s (2007) unpublished dissertation, authors have used the species and subspecies categories interchangeably to describe the three names in the *B. candidus*/*multicinctus*/*wanghaotingi* complex, and the boundaries between species in this group are still subject to controversy (Nguyen et al. 2017; Xie et al. 2018).

During herpetological surveys in Yunnan Province, China, between 2016 and 2019, a series of *Bungarus* specimens were collected from Yingjiang County. These specimens resembled members of the *B. candidus*/*multicinctus*/*wanghaotingi* complex based on morphology, but nested phylogenetically in the same lineage as the specimen CAS 221526 reported by Kuch (2007). Based on multiple evidence including phylogenetical analysis based on three mitochondrial genes, micro-CT scanning, hemipenial morphology, and other morphological data, we evaluated the taxonomic status of these specimens and compared them to all members of the *B. candidus*/*multicinctus*/*wanghaotingi* complex. The results showed that the specimens from Yingjiang County can be distinguished from these taxa, along with all other congeners. We therefore describe these specimens, along with the CAS 221526 specimen from Myanmar, as a new species in this paper.

## Materials and methods

### Sampling

Four individuals of the *Bungarus* were collected from Yingjiang, western Yunnan Province, China. Before preservation, we euthanized these specimens and fixed them in 80% ethanol and then deposited in the Herpetology Museum of Chengdu Institute of Biology, Chinese Academy of Sciences, Chengdu City, Sichuan Province, China (CIB). For comparisons of taxa in *B. candidus*/*multicinctus*/*wanghaotingi* complex, fourteen specimens of *B. m. wanghaotingi* were collected from southern and eastern Yunnan Province and southwestern Guangxi Province, China. Two specimens collected from Saigon, Vietnam, two specimens of *B. m. multicinctus* from Fujian Province and Zhejiang Province were also collected. Additional specimens of the *B. candidus*/*multicinctus*/*wanghaotingi* complex were examined in museum collections for morphological comparisons. A full list of specimens examined can be found in Appendix I. Comparative morphological information on other species was obtained from examined specimens and the literature sources listed in Table 1. Museum acronyms follow Leviton et al. (1985) except for collections that are not included in their list. Chengdu Institute of Biology (**CIB**), Shenyang Normal University (**SYNU**), Naturhistorisches Museum Wien, Vienna, Austria (**NMW**), Muséum National d’Histoire Naturelle, Paris, France (**MNHN**), Rijksmuseum van Natuurlijke Historie, Leiden, the Netherlands (**RMNH**).

To ensure the taxonomic relationships within the *B. candidus*/*multicinctus*/*wanghaotingi* complex, we included specimens of *B. m. multicinctus* near type locality, *B. m. wanghaotingi* near type locality, and monophyletic specimens of *B. candidus* in multiple localities from West Java, Indonesia to Binh Phuoc, Vietnam. Muscle or liver tissues were extracted from specimens before specimens were fixed, preserved in 95% ethanol, and stored at –20 °C.

### Molecular phylogenetic analysis

**Figure 1.**
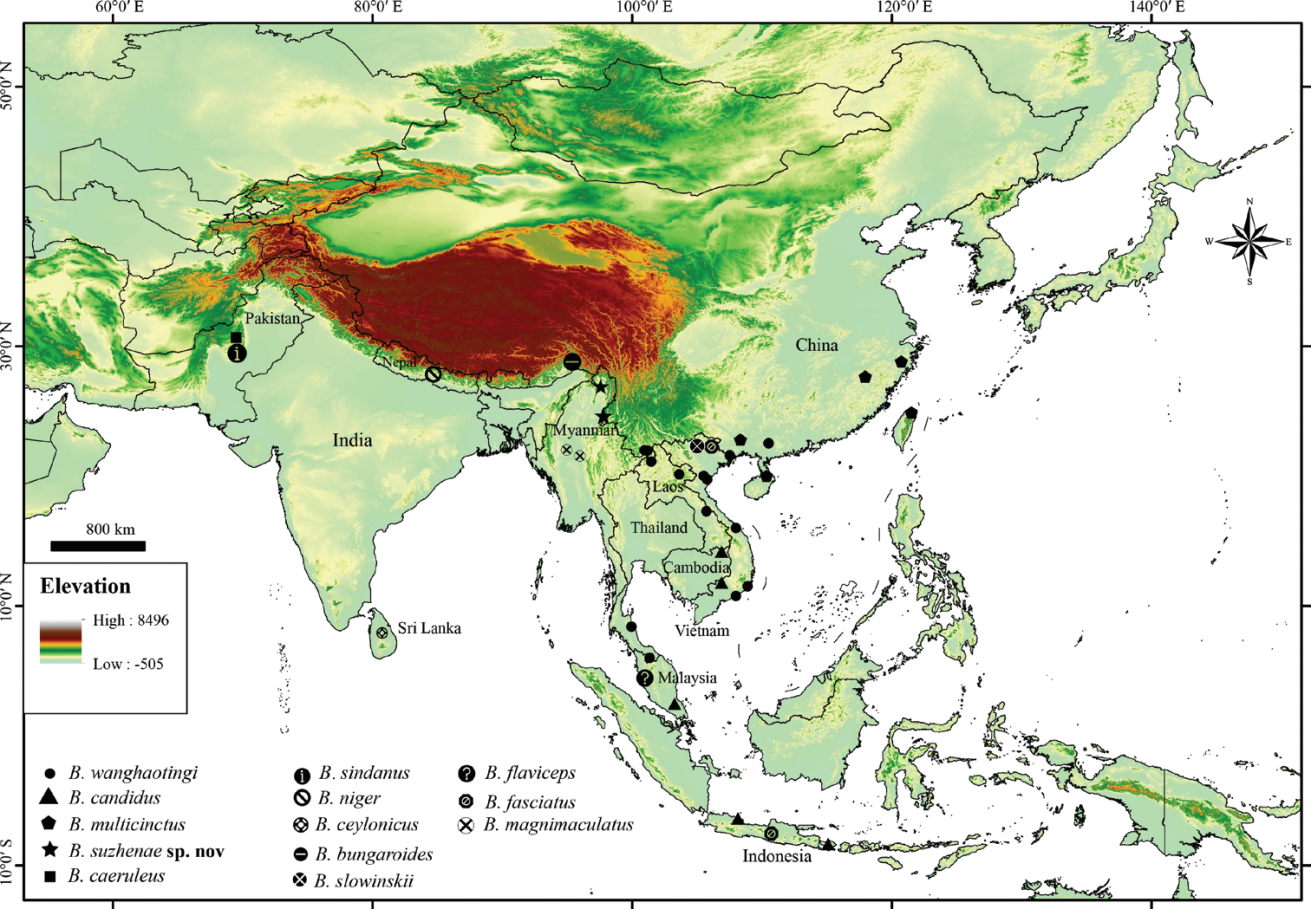
Distribution map of molecular samples localities of *Bungarus* in this study.

Genomic DNA was extracted from muscle or liver tissues using QIAamp DNA Mini Kit (QIAGEN, Hilden, Germany). We sequenced three mitochondrial genes: *cytochrome b* (*cyt b*) (Burbrink et al. 2000), *NADH dehydrogenase subunit 4* (ND4) (Arevalo et al. 1994) and *cytochrome C oxidase 1* (COI) (Che et al. 2012). PCR amplifications were performed in 25 μl reactions (12.5 μl I-5 2×High-Fidelity Master Mix, 10 μl ddH_2_O, 1 μl F-primers, 1 μl R-primers, 0.5 μl DNA template) under the following cycling conditions: initial denaturation for 2 min at 95 °C, 35 cycles with denaturation at 94 °C for 40 s, annealing at different temperatures (48.5 °C for cyt *b* and COI, 56 °C for ND4) for 25 s, extension at 72 °C for 15 s, and final extension for 2 min at 72 °C. PCR products were sequenced by Beijing Qingke New Industry Biotechnology Co., Ltd. Raw trace files for sequences were edited in Geneious 7 (Biomatters Limited, New Zealand) before constructing alignments using MEGA 7 (Kumar et al. 2016). Due to differences in taxon sampling for each gene, we reconstructed separate alignments for phylogenetic analysis. The first was based on a concatenated sequence alignment using cyt *b* and ND4, while the other was based solely on COI. Sequences were uploaded to GenBank under the following accession numbers: MN165132–MN165173. Comparative sequences of available species were downloaded from GenBank (Suppl. material 1: Table S1). Distribution of sequences localities were present in Fig. 1.

Optimal models of sequence evolution of nucleotide substitution were identified by BIC using Partition finder 2.1.1 (Lanfear et al. 2012). We performed maximum likelihood (ML) analysis using RaxML v8 (Stamatakis et al. 2014) and IQ-TREE (Nguyen, et al. 2015) respectively. The first ML analysis was implemented in RaxML v8 (Stamatakis et al. 2014) following GTRGAMMA model with 1000 fast bootstrap replicates to assess node support. We consider bootstrap proportions of 70% or greater as strong support for existence of a clade following Hillis and Bull (1993). The second ML analysis was implemented in IQ-TREE (Nguyen et al. 2015), with Ultrafast Bootstrap Approximation (UFB; Hoang et al. 2017) using 5000 bootstrap replicates to assess node support. Nodes with UFB values of 95 and above were considered significantly supported (Hoang et al. 2017). The best evolution models were shown in supplementary materials (Suppl. material 1: Table S2). Bayesian inference phylogenetic trees were inferred using MrBayes 3.2 (Ronquist et al. 2012). We used a random starting tree and four independent runs with a maximum of 20 million generations each, sampled every 1000. Runs were stopped when the average standard deviation of split frequencies had reached 0.001. The first 25% of each run were discarded as burn-in. Nodes with Bayesian posterior probabilities (BPP) of 0.95 and above were considered well supported (Huelsenbeck et al. 2001).

### Morphological analysis

Measurements of head and head scales were taken with a digital caliper and rounded to the nearest 0.1 mm; snout–vent length and tail length were taken with a measuring tape and rounded to the nearest 1 mm. Terminology and descriptions follow Slowinski (1994), Vogel et al. (2004), and Kuch et al. (2005). Morphometric and meristic characters are abbreviated as follows: total length (TL), from the tip of snout to the tip of tail; snout-vent length (SVL), from the tip of snout to anterior margin of cloaca; tail length (TaL), from posterior margin of cloaca to the tip of tail; ratio of tail length to total length (TaL/TL); head length (HL), from the snout tip to the posterior margin of the mandible; head width (HW) was measured at the widest part of the head on posterior side; head height (HH), at the maximal highest part of the head; the eye horizontal diameter (ED); the eye vertical diameter (VED); distance lower eye margin–edge of the lip (DEL) was measured from the ventral margin of the middle of the eye to the ventral margin of the upper labial below it; the distance from the eye to the nostril (DEN) was measured from the anterior margin of the eye to the posterior margin of the nostril; the dorsal scale rows (DSR) were counted at one head length behind the head, at midbody, and at one head length before the vent; ventral scales (VEN) were counted according to Dowling (1951); half ventrals were counted as one. The enlarged shield(s) anterior to the first ventral were regarded as preventral(s); for subcaudals (SC), first scale under the tail meeting its opposite was regarded as the first subcaudal scale, and the unpaired terminal scute was not included in the number of subcaudals; paired scales on head were counted on both sides of the head and presented in left/right order; supralabials (SL); infralabials (IL) were considered scales and shields that are completely below a supralabial and border the gap between lips. For the number of white bands on the body (BB) and white bands on the tail (TB), incomplete white rings were counted as one. Sex was determined by making a small incision below the vent to visually inspect for the existence of hemipenes. Descriptions of the hemipenes were based on one population of the new species and three populations of the *B. candidus*/*multicinctus*/*wanghaotingi* complex. Hemipenis terminology follows Dowling (1951) and the organs were prepared based on Jiang (2010).

For obtaining information on skeletal morphology, micro-CT scans of skulls were carried out using a 225-kV micro-computerized tomography, developed by the Institute of High Energy Physics (IHEP), Chinese Academy of Sciences (CAS). A total of 720 transmission images were reconstructed into a 2048 × 2048 matrix of 1536 slices using two-dimensional reconstruction software developed by IHEP, CAS. The final CT reconstructed skull images were exported with a minimum resolution of 14.1 (Holotype) and 29.0 (paratype) μm. The dataset of the 3D models included in this study has been uploaded to the online publicly accessible repository ADMorph at http://www.admorph.org/ (Hou et al. 2020).

## Results

### Phylogenetic analysis

The concatenated alignment for cyt *b* and ND4 was 1934 bp in length (1069 + 865 bp, respectively) and contained a total of twelve *Bungarus* species. The COI alignment was 613 bp and contained a total of five taxa. Our results show that the most well-supported cyt *b*-ND4 and COI phylogenetic trees were achieved by using Bayesian Inference (BI), followed by Maximum likelihood RaxML (ML) and IQ-TREE (UFB), respectively.

**Figure 2.**
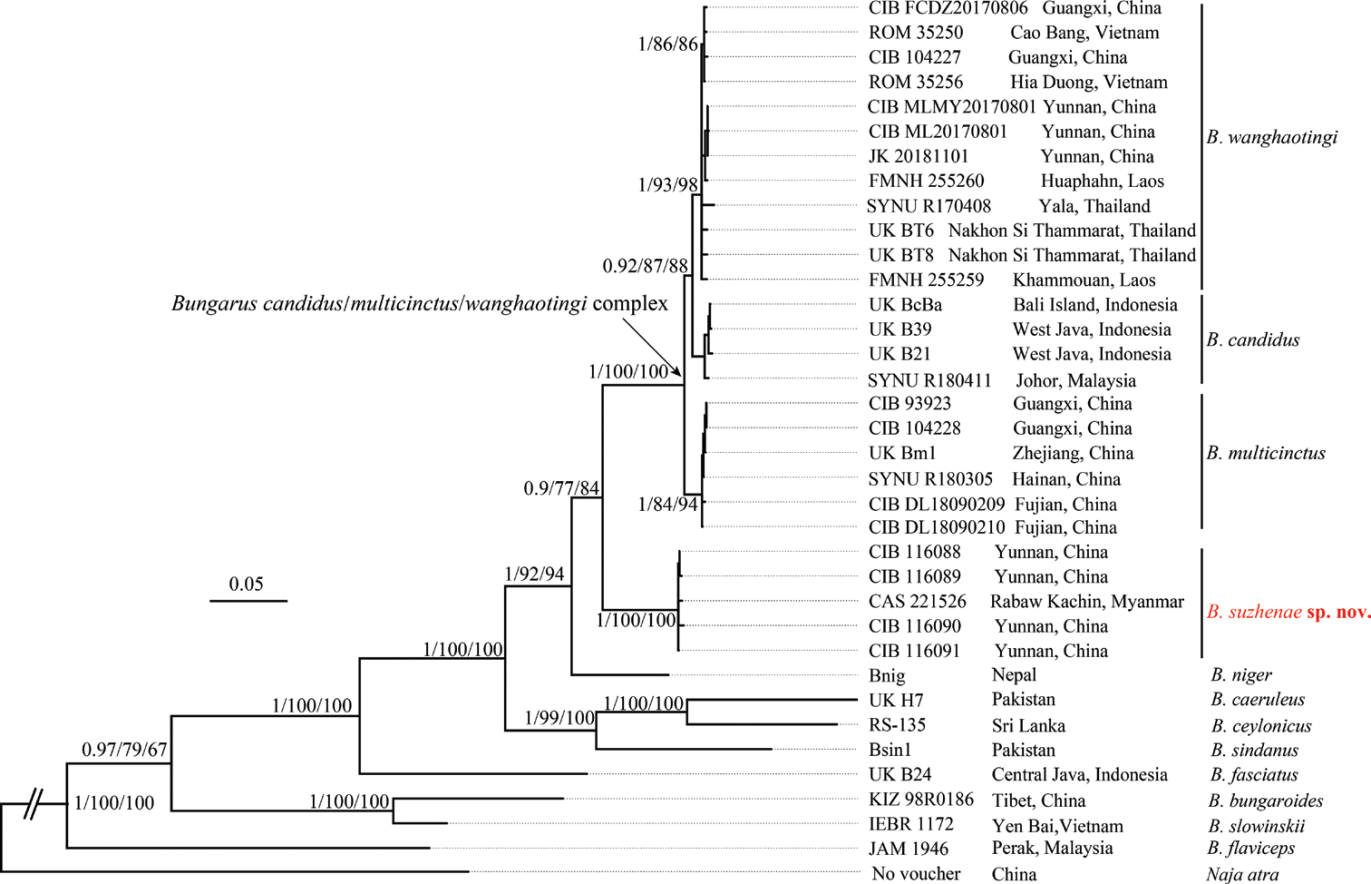
Bayesian inferred tree of the genus *Bungarus* based on combined cyt *b* and ND4 genes fragments. The major clade genetic events BI/ML/IQ posterior probabilities, bootstrap and UFB values were presented (the ones lower than 50 are displayed as “-”).

The topological structures of the combined cyt *b* and ND4 sequences (Fig. 2) concur with an earlier study by Kuch (2007). The new species forms a monophyletic lineage with strong support (BI 1.00/ML 100/UFB 100) sister to the *B. candidus*/*multicinctus*/*wanghaotingi* complex. Within the complex, three strongly supported (BI 0.92/ML 87/UFB 88) monophyletic lineages are identified. The first is *B. wanghaotingi* including specimens from localities near type locality and other specimens from Southern China, Southwestern China, Vietnam, Laos and Southern Thailand; the second is *B. candidus*, including specimens from Java and Bali Island, Peninsular Malaysia; the third is *B. multicinctus*, including specimens near type locality and from other localities in Eastern and Southern China. The two lineages *B. wanghaotingi* and *B. candidus* consist of a clade sister to *B. multicinctus*. The uncorrected *p*-distances of available cyt *b* sequences between the *Bungarus* species are shown in the supplementary material (Suppl. material 1: Table S3). The distances between the new species and its closest congeners in the *B. candidus*/*multicinctus*/*wanghaotingi* complex range from (9.7%–11.6%), similar to the distance between *B. niger* and the *B. candidus*/*multicinctus*/*wanghaotingi* complex (11.0%–12.4%). The genetic distances between the three lineages of the *B. candidus*/*multicinctus*/*wanghaotingi* complex were relatively small, ranging from 1.6% to 3.3%.

**Figure 3.**
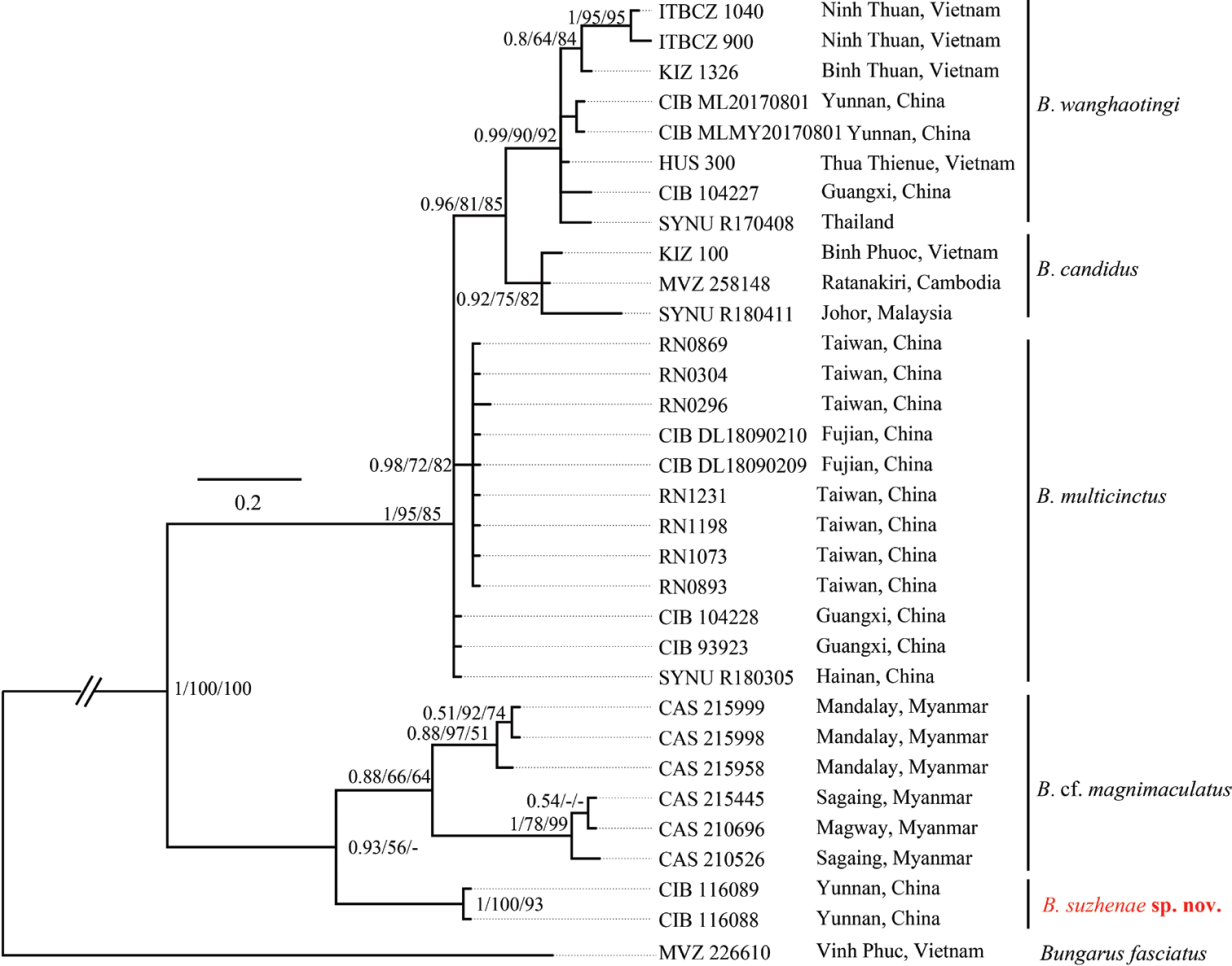
Bayesian inferred tree of the genus *Bungarus* base on COI genes fragments. The major clade genetic events BI /ML/IQ posterior probabilities, bootstrap and UFB values were presented (the ones lower than 50 are displayed as “-”).

In the phylogenetic analyses for the COI alignment (Fig. 3), the new species forms a lineage sister to *B. magnimaculatus* instead of the *B. candidus*/*multicinctus*/*wanghaotingi* complex. The specimens of *B. magnimaculatus* from three localities of Myanmar were formed by two lineages with short branches similar to an earlier study (Nguyen et al. 2017). We regard the two lineages of *B. magnimaculatus* as a species complex. The distinct pairwise genetic distance between these two lineages (2.4%–3.1% for COI) indicates that the species diversity of this species may be underestimated. The topology of the *B. candidus*/*multicinctus*/*wanghaotingi* complex agrees with the concatenated cyt *b*-ND4 gene trees and can be considered to constitute three strongly supported lineages (BI 1.00/ML 95/UFB 85) with very short branch lengths. The uncorrected *p*-distances of COI between the species groups are shown in the supplementary material (Suppl. material 1: Table S4). The new species is separated from the *B. candidus*/*multicinctus*/*wanghaotingi* complex by a distinct distance of 4.4%–5.0%, equivalent to the distance between *B. magnimaculatus* and the *B. candidus*/*multicinctus*/*wanghaotingi* complex (4.4%–6.5%). The high pairwise distances between the new species and its congeners support its recognition as a distinct, independently evolutionary lineage that is not conspecific with any other congeners.

### Morphological analysis

**Figure 4.**
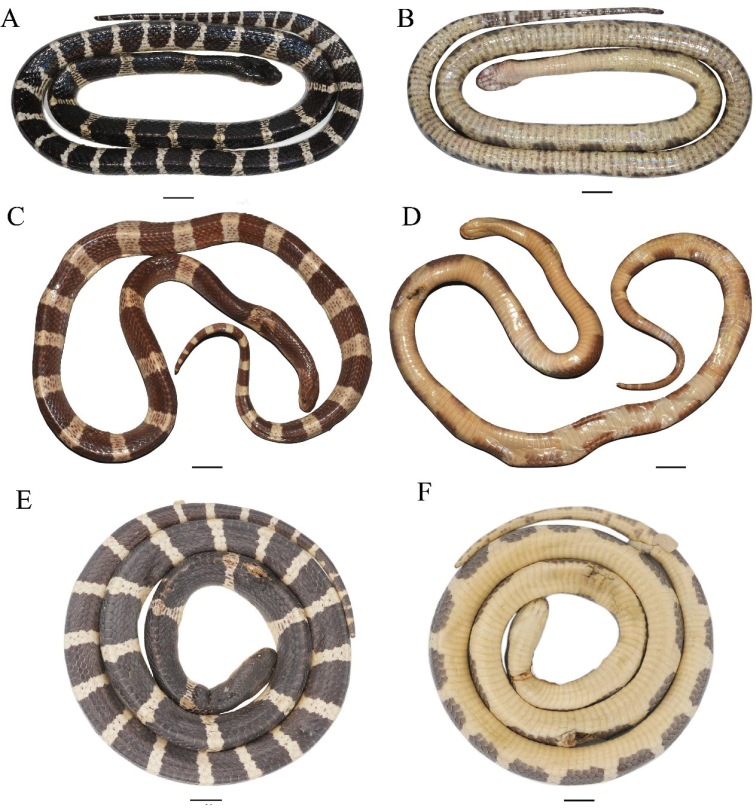
Dorsal (left) and ventral (right) view of adults of the *Bungarus candidus*/*multicinctus*/*wanghaotingi* complex **A, B***Bungarus multicinctus*, adult male, CIB DL2019051701 from Lishui, Zhejiang, China **C, D***B. candidus*, female, NMW 9486:1 from Pelambang, Java **E, F***B. wanghaotingi*, male, CIB MLML20170801 from Mengla, Yunnan, China. Scale bars: 20 mm.

**Figure 5.**
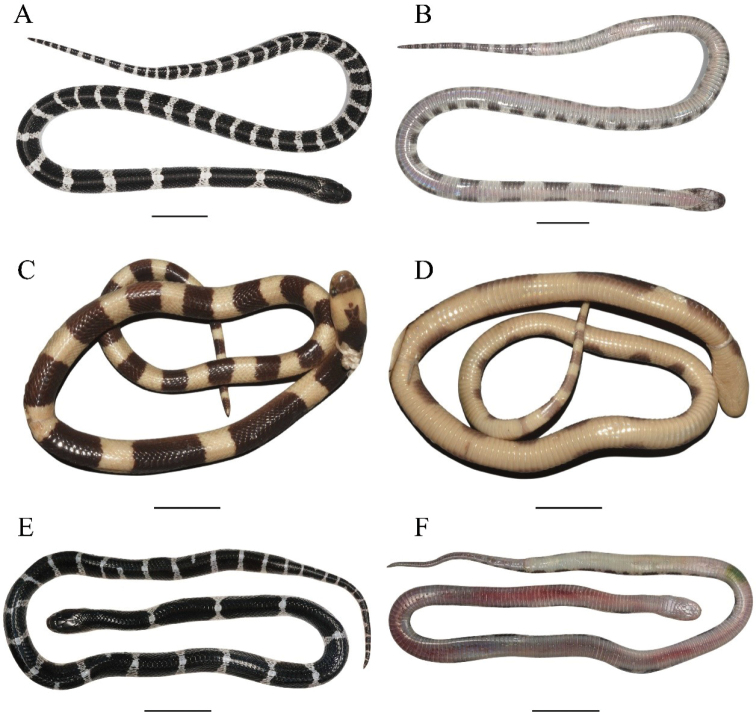
Dorsal (left) and ventral (right) view of juveniles of the *Bungarus candidus*/*multicinctus*/*wanghaotingi* complex **A, B***B. multicinctus*, female, CIB DL18090209 from Fujian, China **C, D***B. candidus*, female, NMW 27730:4 from Tasikmalaya, Java **E, F***B. wanghaotingi*, unknown sex, CIB JCR36 from Jiangcheng, Yunnan, China. Scale bars: 20 mm.

**Figure 6.**
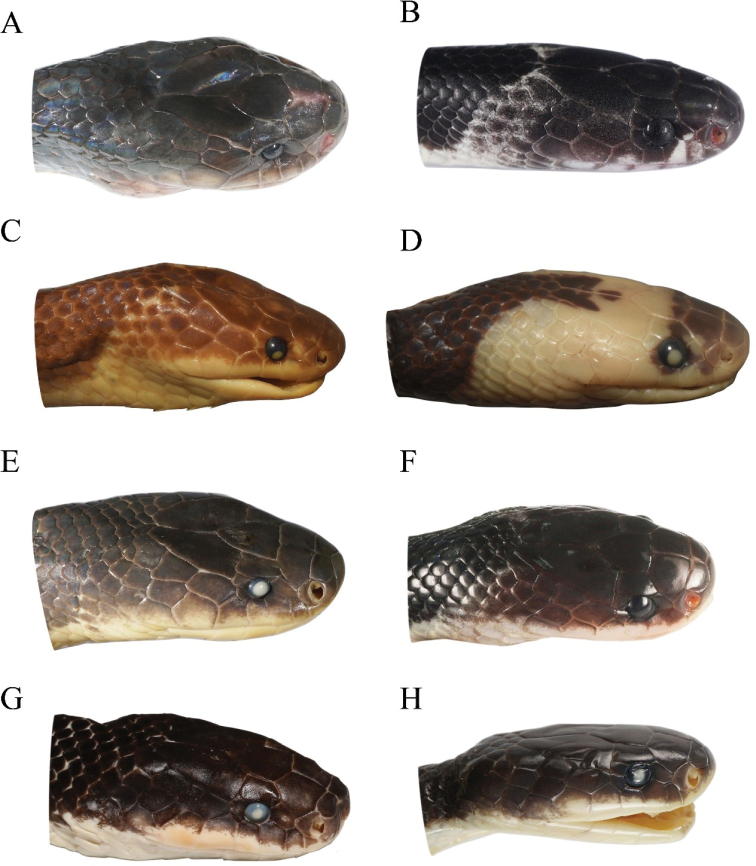
Dorsolateral head view of adults (left) and juveniles (right) of the *Bungarus candidus*/*multicinctus*/*wanghaotingi* complex and *B. suzhenae* sp. nov. **A***B. multicinctus*, adult male, CIB DL2019051701 from Lishui, Zhejiang, China **B***B. multicinctus*, juvenile female, CIB DL18090209 from Fujian, China **C***B. candidus*, adult female, NMW 9486:1 from Pelambang, Java **D***B. candidus*, juvenile female, NMW 27730:4 from Tasikmalaya, Java **E***B. wanghaotingi*, adult male, CIB MLML20170801 from Jiangcheng, Yunnan, China **F***B. wanghaotingi*, unknown sex juvenile, CIB JCR36 from Jiangcheng, Yunnan, China **G***B. suzhenae* sp. nov. adult female, CIB 116090 **H***B. suzhenae* sp. nov. subadult male, CIB 116088.

**Figure 7.**
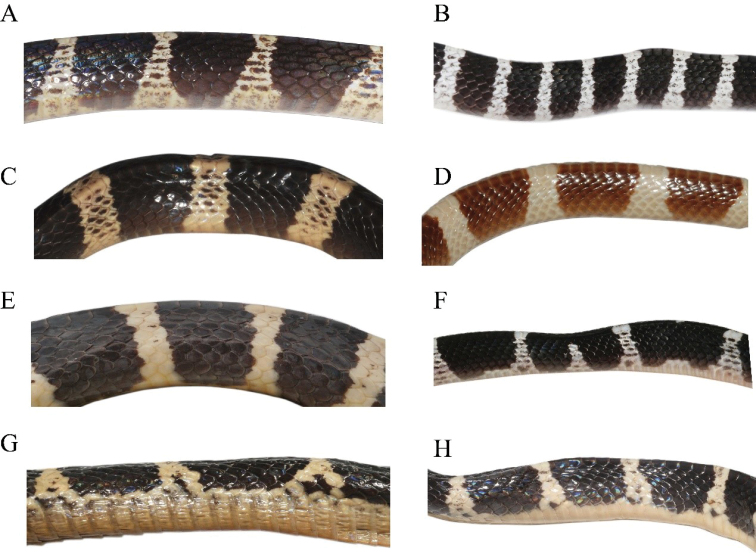
Body patterns of adults (left) and juveniles (right) of the *Bungarus candidus*/*multicinctus*/*wanghaotingi* complex and *B. suzhenae* sp. nov. **A***B. multicinctus*, adult male CIB DL2019051701 from Lishui, Zhejiang, China **B***B. multicinctus* juvenile female, CIB DL18090209 from Fujian, China **C***B. candidus*, adult male, NMW 27711:1 from Bandong, Java **D***B. candidus*, juvenile male, RMNH 11416 from Pelambang, Java **E***B. wanghaotingi*, adult male, CIB MLML20170801 from Mengla, Yunnan, China **F***B. wanghaotingi*, unknown sex juvenile CIB JCR36 from Jiangcheng, Yunnan, China **G***B. suzhenae* sp. nov. adult male, CIB 116089 from Yingjiang, Yunnan, China **H***B. suzhenae* sp. nov. subadult male, CIB 116088 from Yingjiang, Yunnan, China.

**Figure 8.**
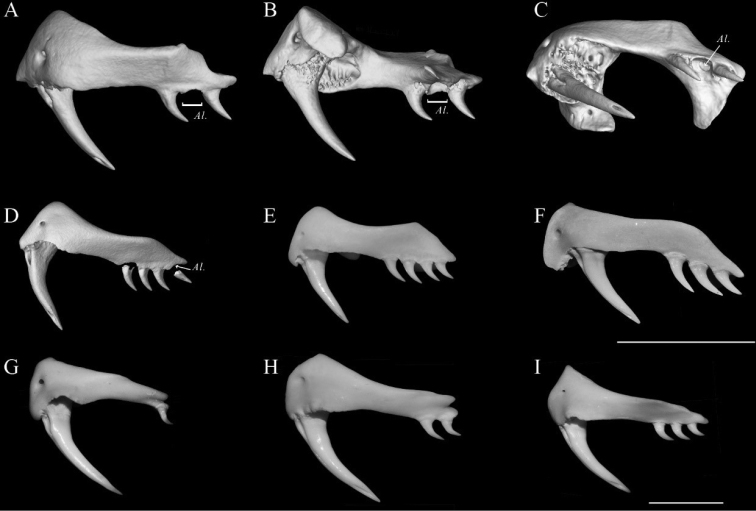
Maxilla morphology of seven members of subfamily Elapidae. Lateral (**A**) lingual (**B**), and ventral (**C**) view of the left maxilla of CIB 116090 (the paratype of *B. suzhenae* sp. nov.), compared with other members of the subfamily Elapinae**D***B. wanghaotingi*SYNU R170408, from Yala, Thailand **E***B. multicinctus*, from Guangdong, China **F***B. fasciatus*, from Guangdong, China **G***Naja atra* from Guangdong, China **H***N. melanoleuca* from Kimpese, Congo **I***Ophiophagus Hannah* from Guangxi, China. Scale bars: 5 mm, note that **H** and **I** had been scaled down at 1/2. Al = alveoli.

**Figure 9.**
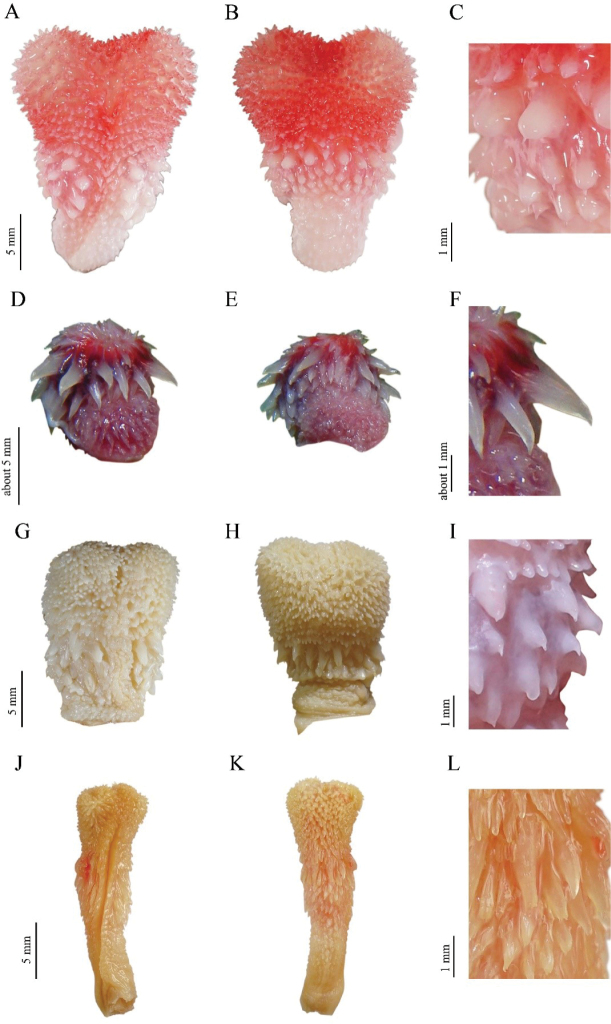
Hemipenial morphology of the *Bungarus candidus*/*multicinctus*/*wanghaotingi* complex and *B. suzhenae* sp. nov. Sulcate view (left), asulcate view (middle), spines (right) **A–C***B. multicinctus*, CIB DL2019051701 from Lishui, Zhejiang, China, body length 993 mm **D–F***B. candidus*, RH06153 from Quang Binh Province, Vietnam, body length 1200 mm **G–I***B. wanghaotingi*, CIB MLML20170801 from Mengla, Yunnan, China, body length 1170 mm **J–L***B. suzhenae* sp. nov., CIB 116089 from Yingjiang, Yunnan, China, body length 1140 mm.

Morphologically, the three taxa of *B. candidus*/*multicinctus*/*wanghaotingi* complex are different from each other in hemipenial morphology, and coloration patterns (morphology of white bands, ventral coloration, and coloration on temporal and lateral neck regions) (Tables 1, 2, Figs 4–9). Thus, we confirm these three monophyletic taxa of *B. candidus*/*multicinctus*/*wanghaotingi* complex as three distinct species: *B. candidus* (Linnaeus, 1758), *B. multicinctus* Blyth, 1860, and *B. wanghaotingi* Pope, 1928.

**Table 2.** Comparison of pattern features and hemipenis morphology in of the *B. candidus*/*multicinctus* complex.

Species	Coloration and patterns					Hemipenis morphology		
	Vertebral scales covered by white bands on middle body	Heads and necks of adults	Heads and necks of juveniles	Ventral surface of body	Ventral surface of tail	SLS	LK	Shape of tips
***B. multicinctus* n = 24**	1.4 ± 0.4, (1.0–2.0)	uniform black	scales on lateral neck dim white edged	white, with dense with brown pigments	dense black bands and patches	papilla-like	weak	rod like, with a distinct boundary with large spines
***B. candidus* n = 18**	3.8 ± 0.6, (3.0–5.0)	temporal area and lateral neck stained white	temporal area and lateral neck creamily white	immaculate white	broad dark crossbands	in shape of fangs	strong	cone, no clear boundary with large spines
***B. wanghaotingi* n = 23**	2.2 ± 0.4, (1.5–2.5) n = 7	uniform black	light brown	immaculate white	a row of small light brown dots on middle	thick, relatively short, most pointy,	weak	larger at the bases, no clear boundary with large spines
***B. suzhenae* sp. nov n = 4**	1.5 ± 0.4, (1.0–2.0)	uniform black	uniform black	immaculate white	immaculate or with small brown dots	in shape of fangs	strong	cone, no clear boundary with large spines

Abbreviations. – SLS (Shape of large spine); LK (Level of keratinization on tips).

The specimens from Yingjiang (Yunnan Province) and Myanmar differ from other species of *Bungarus* in crossbands shape, tail pattern (Figs 4, 5, Table 2), head pattern (Fig. 6), mid-body pattern (Fig. 7), the maxilla teeth (Fig. 8 and Table 3) and hemipenial morphology (Fig. 9, Table 2). Therefore, combining morphological and molecular evidence, we identify those specimens from Yingjiang, Yunnan Province, and Myanmar as a new species.

**Table 3.** Teeth count of some members of subfamily Elapidae in this study.

Species	Maxilla	Palatine	Pterygoid	Dental
***Bungarus suzhenae* sp. nov. (n = 2)**	1+3	10–11	9–10	15–16
***B. candidus***	1+4	/	/	/
***B. wanghaotingi* (n = 1)**	1+4	12–13	10	16–17
***B. multicinctus* (n = 1)**	1+4	12	11	16
***B. fasciatus* (n = 2)**	1+3	13	11–13	17
***Ophiophagus hannah* (n = 3)**	1+3	8–9	10–12	15–16
***Naja melanoleuca* (n = 3)**	1+2	7–9	13–16	15–16
***N. atra* (n = 3)**	1+1	7–8	12–15	14–16
***Sinomicrurus kelloggi* (n = 1)**	1+1	8	4	11
***S. macclellandi* (n = 1)**	1+0	8	9	15

### Taxonomy

#### *Bungarus candidus/multicinctus/wanghaotingi* complex

##### Bungarus candidus

Taxon classificationAnimaliaSquamataElapidae

(Linnaeus, 1758)

B28A12D4-F521-5F33-AC6F-609B5D59AEDA

Figs 4C, D , 5C, D , 6C, D , 7C, D , 9D–F [English name: Blue Krait] [Chinese name: 马来环蛇]

Coluber candidus Linnaeus 1758: 223.
Bungarus candidus – Cantor 1847
Bungarus semifasciatus Boie 1827
Aspidoclonion semifasciatum – Wagler 1828
Bungarus candidus var. semifasciata – Werner 1900
Bungarus javanicus Kopstein 1932 (*fide*Slowinski 1994)
Bungarus candidus – Smith 1943: 416

###### Type locality.

“Indiis” (in error). ***Holotype***: NRM 37 (formerly ZIUS 89).

Typical *B. candidus* possesses following morphological characters based on the examination of 19 specimens from Sumatra and Java, Indonesia; Peninsular Malaysia (Appendix 1): (1) Dorsum of most specimens with 21.4 ± 1.8, (18–26) broad white crossbands, with each band covers 3.8 ± 0.6, (3.0–5.0) vertebral scales on midbody, (Figs 4C, D, 5C, D), uniform black in some populations (Kuch 2007); (2) ventral body immaculate white, without brown pigments (Figs 4C, D, 5C, D); (3) scales on temporal area and lateral neck stained white, contrast with neighbor scales on neck in adults, creamy white in juveniles (Fig. 6C, D); (4) black bands on body large, covering 3–5 vertebral scales on middle body, intruding to white ventral body, ventrals with narrow black edges 1–2 times of outer dorsal scales (Fig. 7C, D); (5) ventral tail with broad dark crossbands (Figs 4D, 5D); (6) posterior maxilla teeth four (n = 9), slightly curved behind, (Table 3); (7) prefrontal suture 1.4–2.4 (n = 17) times the length of internasals suture; (8) VEN = 209–224 (n = 18), NSC = 41–50 (n = 17).

The hemipenes of *B. candidus* is described based on photos of a male (Fig. 9D–F, collecting No. RH06153, total length 120 cm) from Phong Nha-Ke Bang National Park Administration, Quang Binh Province, Vietnam, by Ralf Hendrix. This specimen was tentatively identified as *B. candidus* by the presence of (1, 2, 4–8) characters in the former morphological description. The hemipenis was partially everted, with large spines present on the medial portion of the organ at the position of the first subcaudal scale; smaller spinous calyces present near the base and another spinous zone present posterior from the row of larger spines. Spinous calyces along organ all elongated, robust at bases and gradually tapering to a tip without distinct bordering. Tips of spines strongly keratinized, semitransparent when fresh, bent towards the base of hemipenes. Sulcus not shown in the photos.

Distribution. This species is known from following localities based on specimens examined and/or DNA sequences data: Java and Sumatra Island, Indonesia; Peninsular Malaysia; Cambodia; Central and Southern Vietnam.

##### Bungarus multicinctus

Taxon classificationAnimaliaSquamataElapidae

Blyth, 1860

BC2E5450-8ED9-58C1-B039-693FB29E842E

Figs 4A, B , 5A, B , 6A, B , 7A, B , 8E , 9A–C [English name: Many-banded Krait] [Chinese name: 银环蛇]

Bungarus multicinctus BLYTH 1860: 98.
Bungarus semifasciatus Günther 1858: 221 (not of Boie)
Bungarus candidus var. multicinctus – Boulenger 1896: 369

###### Type locality.

Likely Amoy (now Xiamen, Fujian Province, China), possibly Formosa (Taiwan, China). ***Holotype***: lost (*fide*Smith 1943; Nguyen et al. 2009)

This species was described based on one specimen from Amoy (Blyth 1860). The following description is based on 24 examined specimens from Southern China (Appendix 1): (1) narrow white dorsal crossbands 39.3 ± 4.7 (31–50), with each 1.4 ± 0.4 (1.0–2.0) vertebral scales long at midbody (Tables 1, 2, Figs 4A, B, 5A, B); (2) ventral body white scattered with dense brown pigment on adults (n = 19) (Fig. 3B), indistinct on some juveniles (Fig. 5B); (3) scales on neck and head of adults uniform black, scales on lateral neck behind parietals for immatures indistinctly edged with white (Fig. 6A); (4) moderately wide black bands on body (3–4 vertebral scales wide) intruding to ventrals for 1.2 to 2 times the width of outer dorsal scales (Fig. 7A); (5) ventral surface of tail with dense black bands and patches (Figs 4B, 5B); (6) posterior maxilla teeth four, distinctly curved backwards (Fig. 8E and Table 3); (7) fangs distinctly curved posteriorly (Fig. 8E); (8) prefrontal suture 1.5–2.5 (n = 17) times length of the internasal suture; (9) VEN 196–236 (n = 24), NSC 38–58 (n = 23).

Hemipenes description based on a sequenced male (Fig. 9A–C, CIB DL2019051701, SVL 993 mm) from Lishui, Zhejiang, China. Hemipenes reaches 9^th^ subcaudal, bilobed near apex. Base of the organ covered with tiny soft basal hooks, medial portion spinous and apex fully calyculate, with the area between the calyculate zone and spinous zones poorly defined. Most spines on the organ thick, papilla-shaped and blunt, each surmounted by minute, sharp, spine-like tip pointing towards base of hemipenes. The tips are weakly keratinized, concentrated in the shape of short bars; width consistent throughout organ, with a distinct boundary along the region between the main part of papilla-shaped spines and its tips. The morphology of large spines on hemipenes are similar to the morphology of a male from Changsha, Hunan Province, China, which was described by Pope (1935).

*B. multicinctus* differs from *B. candidus* by having (1) more white bands on the body (31–50, n = 24 vs. 18–26, n = 19) that are narrower in length (1–2 times of length of vertebral scales on middle body vs. 3–5 times); (2) different adult ventral surface (dense brown pigment vs. immaculate white); (3) different coloration of scales on the temporal and lateral neck regions (uniform black in adults and dim white edged in immatures vs. stained white in adults and creamy white in juveniles); (4) shape of the spines on the hemipenis (blunt, papilla-like vs. large spines that are sharp and fang-shaped); (5) and by the degree of keratinization of the hemipenial spines (tips of large spines not strongly keratinized, in shape of short bars, with a distinct boundary with the body of large spines vs. tips of large spines strongly keratinized, gradually wider towards base of large spines).

Distribution. This species is known from the following provinces in China based on specimens examined and/or DNA sequences data: Zhejiang, Fujian, Anhui, Guangdong, Guangxi, Hainan, Taiwan, Chongqing and Guizhou. It is also reported from Hunan Province (Pope 1935).

##### Bungarus wanghaotingi

Taxon classificationAnimaliaSquamataElapidae

Pope, 1928

D19369E0-6EB7-53E2-BC03-2E318DF6FA96

Figs 4E, F , 5E, F , 6E, F , 7E, F , 8D , 9G–I [English name: Wang’s Krait] [Chinese name: 云南环蛇]

Bungarus multicinctus wanghaotingi Pope 1928: 3.
Bungarus multicinctus wanghaotingi – Mell 1929; Zhao et al. 1998; Zhao 2006
Bungarus wanghaotingi – Leviton et al. 2003

###### Type locality.

Yuankiang, Yunnan, China. ***Holotype***: AMNH 35230.

The typical populations of this species possess the following characters based on 16 examined specimens from Yunnan and Guangxi, China (Appendix 1), squamation data and body measurements of ten specimens from Yunnan, China (Yang and Rao 2008) and the holotype (Pope 1928): (1) 25.1 ± 3.2 (18–33, n = 27) narrow white dorsal body bands, 1.5–2.5 (n = 16) vertebral scales long at midbody (Yunnan population, n = 7; wider on specimens from Thailand) (Figs 4E, 5E); (2) ventral surface immaculate (Figs 4F, 5F); (3) scales on neck and head uniform black in adults, light brown in juveniles (Fig. 6E, F); (4) moderately elongate black bands on body (3.5–6.0 vertebral scales long) intruding to ventrals for 0.5 to 1.5 times of length of outer dorsal scales (Fig. 7E, F); (5) ventral tail white with one row of small light brown dots in the middle of the subcaudals (Figs 4F, 5F); (6) posterior maxilla teeth four, slightly folding backwards (Fig. 8D and Table 3); (7) fangs distinctly curved (Fig. 8D); (8) prefrontals suture 1.2–2.5 (n = 10) times the length of internasals suture; (9) VEN = 209–259 (n = 23), NSC = 32–64 (n = 22).

The hemipenes (Fig. 9G–I) are described based on the sequenced adult male specimen CIB MLMY20170801 (SVL 1170 mm) from Mengla, Yunnan Province and one subadult male CIB DL2019051401 from, Yunnan Province, China; hemipenes reach 9^th^ subcaudal, bilobed near apex, can be divided into three zones similar to *B. multicinctus*, the line of demarcation between the calyculate zone and the spinose zone is poorly defined; large spines thick, relatively short, mostly pointy, gradually thinning from the base to the tip; tips of large spines weakly keratinized, degree of keratinization highest at base, not in shape of short bars and not having a distinct boundary with main body of large spine.

*B. wanghaotingi* (typical populations from China) differs from *B. multicinctus* by having (1) fewer white bands on body; (2) ventral colouration of the body (immaculate vs. scattered with dense brown pigments in adults) (Fig. 5A, B); (3) coloration of the ventral surface of tail (immaculate or with dots vs. broad dark bands or patches); (4) the morphology of large spines on the hemipenes (large spines on hemipenes mostly pointy vs. papilla-like in shape and blunt); (5) the shape of the large spines on the hemipenes (without a distinct boundary with main body of large spines vs. with a distinct boundary); (6) fang shape (less distinctly curved vs. distinctly curved) (Fig. 7); and (7) posterior maxilla teeth less folding behind (Fig. 7).

*B. wanghaotingi* (typical populations from China) differs from *B. candidus* by having (1) narrower white bands in most specimens; (2) scales on neck and dorsal head uniform black in adults, light brown in juveniles vs. stained white, contrasting with neighbor scales on neck in adults, creamy white in juveniles; (3) ventral tail immaculate or with dots, rather than broad dark bands; (4) large spines on hemipenes relatively short, and weakly keratinized (vs. very elongated, and strongly keratinized).

Distribution. This species is known from the following localities based on specimens examined and/or DNA sequences data: Southern Yunnan, Southern Guangxi, China; Southern, Central and Northern Vietnam; Northern and Central Laos; Southern Thailand.

##### Bungarus suzhenae sp. nov.

Taxon classificationAnimaliaSquamataElapidae

7DA0013F-DE48-59FC-80AC-8C36B2951EA6

http://zoobank.org/8F3B0FA6-9B11-4CE3-AAD6-926D159D5220

Figs 6G, H , 7G, H , 8A–C , 9J–K , 10 , 11 , 12 , 13

Bungarus multicinctus multicinctus – Yang and Rao 2008, specimen from Yingjiang, Yunnan, China

###### Type material.

***Holotype*.**CIB 116088 (Fig. 9), subadult male, collected from a road through rice fields in Yingjiang County, Yunnan Province, China (97.584451, 24.662632, 922 m A.S.L), by Ding Li, in 2017. The holotype was a victim of roadkill and was fixed and stored in 80% ethanol.

***Paratypes*.** One adult male CIB 116089 (24.466941°N, 97.648691°E, 934 m A.S.L), one adult female CIB 116090 (24.634715°N, 97.762291°E, 1559 m A.S.L), one sub-adult male CIB 116091 from Yingjiang County (24.560296°N, 97.827170°E, 798 m A.S.L). The specimens were preserved in 80% ethanol.

###### Diagnosis.

**Figure 10.**
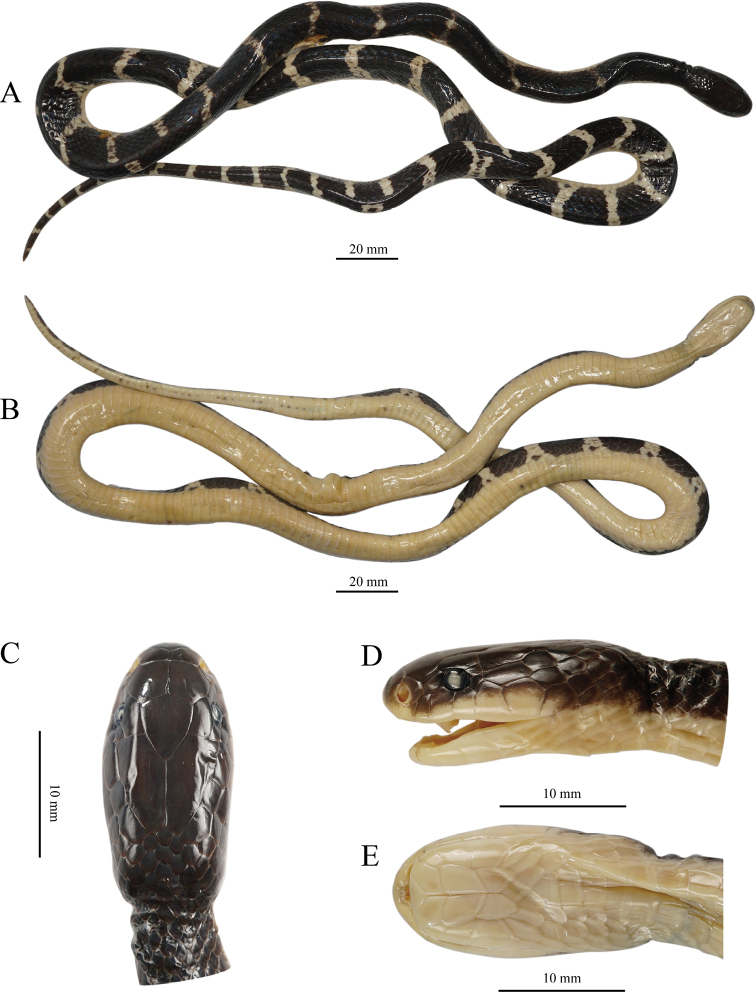
Holotype of *Bungarus suzhenae* sp. nov. (CIB 116088) **A** dorsal view of body **B** ventral view of body **C** dorsal view of head **D** left lateral view of head **E** right lateral view of head.

**Figure 11.**
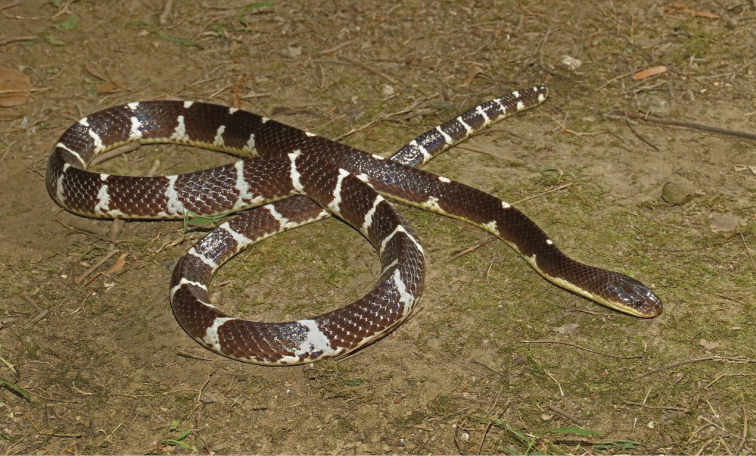
Paratype of *Bungarus suzhenae* sp. nov. in life (Adult female CIB 116090).

**Figure 12.**
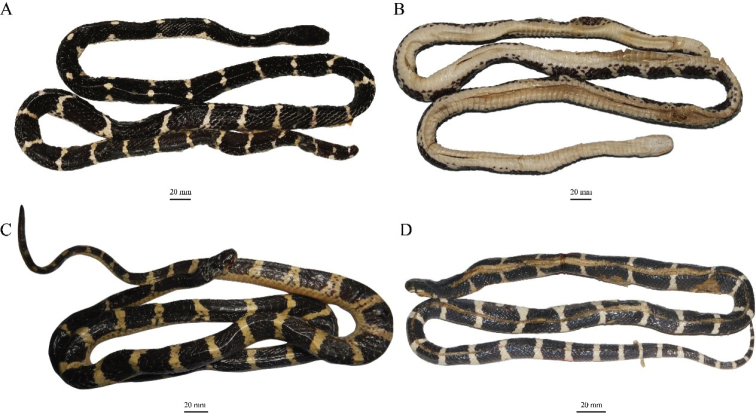
Paratypes of *Bungarus suzhenae* sp. nov. in preserve **A** dorsal and ventral **B** view of adult female CIB 116090 **C** dorsal view of adult male CIB 116089 **D** dorsal view of juvenile male CIB 116091.

Assigned to genus *Bungarus* based on the presence of a row of enlarged, hexagonal scales on the vertebral scale row, enlarged prezygapophyseal accessory process and relatively high neural spine (Slowinski 1994). The new species differs from its congeners by having a combination of the following characters: (1) posterior maxilla teeth three, slightly curved behind (Fig. 8A–C); (2) fangs feebly curved; (3) dorsal scales in 15 rows; (4) ventrals 220–229 (n = 4); (5) subcaudals undivided, 51–54 (n = 3); (6) anterior chin shields larger than the posterior ones (Fig. 10E); (7) prefrontal suture 2.7–3.4 (n = 3) times length of internasal suture (Fig. 10C, D); (8) adult and subadult heads uniform black (Figs 10–12); (9) dorsal body color black, with 39.3 ± 4.7 (26–38) white narrow bands present on midbody, covering 1.5 ± 0.4 (1.0–2.0) vertebral scales; (10) ventral surface uniform white, underside of tail white with tiny brown dots in the middle or immaculate (Figs 10–12); (11) ventral scales connected with the black bands of the dorsal body by small dark patches in lateral view, patches smaller than half the width of a dorsal scale; (12) tail relatively long, TaL/TL = 0.136–0.150 (n = 3); (13) hemipenes reaching 7^th^ subcaudal; (14) large, elongated and pointed spines on hemipenes, in fang-shaped (Fig. 9J–L); (15) tips of the large spines strongly keratinized, without distinct boundary with the main body of large spines.

###### Comparison.

Comparisons of *Bungarus suzhenae* sp. nov. with other *Bungarus* species are shown in Table 1. *Bungarus suzhenae* sp. nov. differs from *B. flaviceps* by: (1) dorsal scales in 15 rows (vs. 13 rows); (2) dorsal body and tail black with white bands (vs. body black with or without light vertebral and paraventral stripes, tail bright red); (3) head uniform black (vs. head red or yellowish-tan).

*Bungarus suzhenae* sp. nov. differs from *B. fasciatus* by: (1) subcaudal scales 51–54 (n = 3) (vs. 23–39, n = ?); (2) dorsal body black with white bands (vs. with broad yellow rings between the dark rings); (3) dorsal head uniform black (vs. with V-shaped marking on the posterior of the head).

*Bungarus suzhenae* sp. nov. differs from *B. bungaroides* by: (1) subcaudals undivided (vs. divided); (2) Dorsum with 26–38 white bands (vs. 40–60 narrow white rings composing of small white spots); (3) ventral body uniform white (vs. blackish with irregular yellowish white pattern in every 3 to 4 scale intervals).

*Bungarus suzhenae* sp. nov. differs from *B. slowinskii* by: (1) subcaudals undivided (vs. divided); (2) anterior chin shields larger than the posterior chin shields (vs. anterior chin shields similar with posterior chin shields); (3) dorsal head uniform black (vs V-shaped marking present on head); (4) dorsal body and tail with black bands, ventral body uniform immaculate yellowish-white (vs. body with pattern of dark and white rings).

*Bungarus suzhenae* sp. nov. differs from *B. ceylonicus* by: (1) subcaudal scales 51–54 (n = 3) (vs. 34–40, n = ?); (2) ventral body uniform immaculate yellowish white (vs. ventral body with broad dark crossbands).

*Bungarus suzhenae* sp. nov. differs from *B. lividus* Cantor, 1839 by: (1) vertebral scales distinctly enlarged (vs. only slightly enlarged on the anterior body); (2) subcaudal scales 51–54 (n = 3) (vs. 41, n = 1); (3) dorsal body black with white bands (vs. no bands or rings or with narrow white rings).

*Bungarus suzhenae* sp. nov. differs from *B. niger* by: (1) dorsal body black with white bands (vs. no bands or rings on body) (Wall 1908); (2) tail relatively longer (TaL/TL = 0.136–0.150 n = 3 vs. 0.132 n = 1).

**Table 4.** Main characters and measurements of *Bungarus suzhenae* sp. nov.

Character	CIB 116088	CIB 116089	CIB 116090	CIB 116091
**Sex**	M	M	F	M
**DSR**	15/15/15	15/15/15	15/15/15	15/15/15
**VEN**	221	229	222	220
**SC**	53	54	11+	51
**SL**	7/7	7/7	7/7	7/7
**IL**	7/7	7/7	7/7	7/7
**BB+TB**	38+12	34+12	34+3+	26+9
**SVL**	620	1140	1310	700
**TaL**	109	180	/	113
**HL**	21	39	30.2	/
**HW**	12.3	15.5	19.4	/
**HH**	8.7	12.8	14.2	/
**ED**	9.3	10.5	14.6	/

Abbreviations. – See in Material and methods. Note: CIB 116088 is holotype, and other three is paratypes. The tail of CIB 116090 is incomplete. The head of CIB 116091 was flattened by roadkill.

*Bungarus suzhenae* sp. nov. differs from *B. magnimaculatus* by: (1) more subcaudal scales (51–54 n = 3 vs. 40–48); (2) dorsum with 26–38 white bands, narrower than black bands in between (vs. 11–14 broad, white crossbars, as wide as the black interspaces).

*Bungarus suzhenae* sp. nov. differs from *B. andamanensis* Biswas & Sanyal, 1978 by: (1) more ventral and subcaudal scales (220–229 n = 4 and 51–54 n = 3 vs. 192–197 n = 4 and 45–47 n = 4); (2) a shorter tail (TaL/TL = 0.136–0.150 n = 4 vs. 0.155–0.16 n = 4); (3) dorsum with 26–38 white bands (vs. 44 white linear arches or bars, mottled with brown); (4) head uniform black (vs. head is chocolate); (5) ventral body uniform white (vs. anterior and lateral margin of ventral scales tinged with brown).

*Bungarus suzhenae* sp. nov. differs from *B. sindanus* by: (1) fewer dorsal scale rows (15 vs. 17); (2) dorsal body colouration (black with white crossbands, and bands mostly complete vs. black with crossbands formed by series of white spots and interrupted).

*Bungarus suzhenae* sp. nov. differs from *B. walli* Wall, 1907 by: (1) fewer dorsal scale rows (15 vs. 17 rows); (2) dorsal body coloration (black with white crossbands, and bands mostly complete vs. body black above with crossbands formed by series of white spots and interrupted); (3) a higher number of ventral scales (220–229 n = 4 vs. 198–207 n = 8).

*Bungarus suzhenae* sp. nov. differs from *B. persicus* Abtin, Nilson, Mobaraki, Hooseini & Dehgannejhad, 2014 by: (1) fewer dorsal scale rows (15 vs. 17); (2) fewer ventral scales (220–229 n = 4 vs. 236–238 n = 2); (3) loreal plate absent (vs. present); (4) dorsal body coloration (black with white bands vs. body with crossbars ending in pairs of small rectangular whitish dots or short crossbars).

*Bungarus suzhenae* sp. nov. differs from *B. caeruleus* by: (1) dorsal body coloration (black with white crossbands, and bands mostly complete vs. narrow transverse white streaks or with small white spots); (2) white bands not in pairs (vs. at least some white bands occurring in pairs).

*Bungarus suzhenae* sp. nov. is morphologically most similar to phylogenetically closest congeners in *B. candidus*/*multicinctus*/*wanghaotingi* complex. However, it differs from the latter by multiple morphological characters. See hemipenis and maxilla comparisons in Tables 2, 3. The new species differs *B. multicinctus* by: (1) fang shape (less distinctly curved vs. distinctly curved); (2) lesser posterior maxilla teeth (three vs. four); (3) relatively longer prefrontals suture (length 2.7–3.4 times of internasals suture n = 3 vs. 1.3–2.3 times, n = 16); (4) ventral body coloration in adults (immaculate white, n = 4 vs. white scattered with dense brown pigments, n = 19); (5) black bands on body (large, length 4–7 times of vertebral scales on middle body, not reaching ventrals or just stained the edges of it, ventrals with black edges smaller than half of outer dorsal scales vs. black bands on body moderate, length 3–4 times of vertebral scales, intruding to ventrals for 1.2 to 2 times of width of outer dorsal scales); (6) ventral tail colouration (white with tiny brown dots in the middle or immaculate vs. with dense black bands or patches); (7) relatively shorter hemipenis (reaching 7^th^ subcaudal vs. 9^th^ subcaudal); (8) shape of large spines on hemipenes (elongated, fang-shaped, pointy vs. papilla-like and blunt); (9) tips of large spines on hemipenis (strongly keratinized, without distinct boundary with the main body of large spines vs. weakly keratinized, in shape of short bar, with a distinct boundary with main body of large spine).

*Bungarus suzhenae* sp. nov. differs *B. candidus* by: (1) fewer posterior maxilla teeth (three vs. four); (2) white bands on dorsal body more and narrower (26–38 white bands on dorsal body, width covering 1.0–1.5 vertebral scales on middle dorsum, n = 4 vs. 19–26 white bands on dorsal body, width covering 3.0–5.0 vertebral scales, n = 18); (3) prefrontal suture relatively longer (2.7–3.4 times length of internasals suture, n = 3 vs. 1.4–2.4 times, n = 17); (4) coloration on the upper head surface and neck (uniform black on adults and juvenile vs. temporal area and lateral neck light brown in adults, lateral necks and dorsal head posterior to eyes of immatures creamy white; (5) ventral tail colouration (white with tiny brown dots in the middle or immaculate vs. with broad dark crossbands); (6) black bands on body (not intruding to ventral body, ventrals with narrow black edges smaller than half of outer dorsal scales vs. intruding to the ventral body, narrow black edges on ventrals with width 1–2 times of outer dorsal scales).

*Bungarus suzhenae* sp. nov. differs from typical *B. wanghaotingi* by: (1) slightly curved fangs (slightly curved and arc-like vs. distinctly curved); (2) fewer posterior maxilla teeth (three vs. four); (3) shorter hemipenis (reaches 7^th^ subcaudal vs. 9^th^ subcaudal); (4) shape of large spines on hemipenis (elongated, fang-shaped (vs. relatively short and blunt); (5) the degree of keratinization of the large hemipenial spines (strongly keratinized vs. weakly keratinized).

###### Description of holotype.

(Fig. 10). Subadult male. Head relatively long, length 21.0 mm, maximal head width at anterior temporals 12.3 mm; maximal head height 8.7 mm, head 1.7 times longer than wide, distance between eyes 9.3 mm. Body length 620 mm; tail complete, 109 mm; total length 729 mm.

###### Body scalation.

Ventrals 221, preventrals 3, anterior edge of first ventral starting at level of oral rictus; azygous scale immediately anterior to cloacal scale, half in width of the ventrals. Cloacal plate undivided. Subcaudals 53 undivided, tail complete. Dorsal scales smooth, in 15–15–15 rows; vertebral scales distinctly enlarged, largest and hexagonal at midbody, slightly wider than long.

###### Head.

Cephalic scales smooth. Rostral near Λ-shaped, width 1.6 times of height visible from above. Nasals large, constricted and divided into one prenasal and one postnasal on both sides at border with internasals and first supralabial, prenasals irregular-shaped while postnasals crescent-shaped. External nares large, vertical oval-shaped, slightly smaller than eye diameter. Postnasal-preocular suture short and straight. Preocular hexagonal, bordered by third and fourth supralabials. Internasals two, 1.1 times wider than long, in contact with rostral, prenasals and postnasals, preoculars, and prefrontals. Prefrontals large, slightly wider than long; internasals suture short, prefrontals suture length 2.9 times of internasals suture and not aligned with latter. Frontal shield-shaped, pointing posteriorly, 1.3 times longer than wide, bordered by prefrontals, supraoculars and parietals; anterior suture of frontal pointed toward prefrontal suture, dividing posterior ends of prefrontals; supraoculars small, 1.7 times longer than wide, in contact with preoculars, upper postoculars, prefrontals, frontal and parietals. Parietals large and long, distance between end of parietals to preoculars 1.5 times the length of frontal; bordered by frontal, supraoculars, upper postoculars, one anterior temporal and two upper posterior temporals on each side, and three smalls nuchal scales on posterior margins. Posterolateral margins of parietals bordered by 1/1 enlarged elongate scales that anteriorly contact upper posterior temporals. Posterior extension of parietals pointed, divided in the middle by one of those three small dorsal scales. Preoculars 1/1, long hexagon, bordering with postnasal, second and third supralabials, prefrontal, and supraocular. Eyes small, oval, horizontal diameter 2.3 mm, vertical diameter 1.9 mm. Postoculars 2/2; relatively small with half size of preoculars; each lower postocular bordered by fourth and fifth supralabials, orbit, anterior temporal, upper postocular; each upper postocular bordered by lower postocular, orbit, supraocular, parietal but not anterior temporal. Anterior temporals 1/1, long and hexagonal, length 2.9 times of width; each bordered by fifth and sixth supralabials, lower postocular, parietal, posterior temporals. Posterior temporals 2/2, bordering parietals, anterior temporals, sixth and seventh supralabials, and enlarged elongate scales bordering posterolateral margin of parietals. Supralabials 7/7, the third and fourth supralabials forming lower margin of orbit; first supralabials small, triangular, with pointed extension behind, not reaching preoculars, 1.4 times higher than wide; other supralabials in different subpentagonal shapes; second supralabials long and pentagonal-shaped, larger than the first, 1.8 times higher than wide; third supralabials larger than the former two, and the fourth, 1.5 times higher than wide; the fourth supralabials more or less rectangular, 1.6 times higher than wide; fifth and sixth supralabials are among the two largest, both 1.1 times higher than wide and similar in size, but fifth supralabials wider at lower part while the sixth supralabials is wider at the upper part; seventh supralabials height equal to width. Mentals moderate, width slightly shorter than width of rostral, triangular, bordering first infralabials, mental groove distinct. Infralabials 7/7 first infralabials pentagonal-shaped, long and narrow, in broad contact behind the mental and anterior chin shields; second infralabials in form of a square, half size of the first; the third and fourth enlarged; first, second, and third infralabials in broad contact with anterior chin shields, fourth infralabials in broad contact with posterior chin shields. Anterior chin shields larger than the posterior chin shields, the two pairs of chin shields in form of butterfly wings; anterior chin shield suture 3.5 times the length of the posterior chin shield suture; posterior chin shields 1.6 times longer than wide, bordered by anterior chin shields, fourth infralabials, 2/2 sublabials, and three gulars. Four gulars between first ventral and posterior most extension of each posterior chin shield; one gular and three preventrals between first ventral and suture of posterior chin shields, preventrals wider than half of first ventrals, gradually larger from first preventral to third.

###### Coloration in preservative.

Dorsal surface of head, upper part of sides of the head, including upper part of supralabials, uniform black; lower half of head, including lower part of supralabials and rostral yellowish-white; ventral head uniform yellowish-white; iris dark black.

Dorsal body black with 38 white narrow crossbands (including incomplete bands). White bands on body scattered with tiny dark patches. Length of bands 1.0 to 2.0 times vertebral scales (average 1.2 ± 0.2), bands widening on flanks before joining the ventral surface, which is uniform white. 10 out of 38 bands incomplete, only present on one side of the dorsal body. First band starts at the 13^th^ ventral, nine vertebral scales between first and second band; following bands gradually denser and brighter, three vertebral scales between 37^th^ and 38^th^ band. Most bands wider on outer row of dorsal scales, a dark spot present at junctions where the white bands meet the ventrals; black bands on body wide, covering 5–6 vertebral scales on middle body, not intruding to venter, ventrals with narrow black edges smaller than half of lateral dorsal scales. Venter immaculate yellowish-white, lateral edges of ventrals between dorsal white bands black.

Dorsal surface of tail black; 12 immaculate white bands present on dorsal part, width about equal to the width of one vertebral. Ventral portion of tail yellowish white, 23 of intermittent subcaudals with small brown dots; subcaudals between white bands margined with brown laterally (Fig. 10).

###### Variation.

Paratypes largely resemble the holotype in scalation and color but differ in the following characters: upper postoculars of one adult male (CIB 116090) and one subadult male (CIB 116091) bordered by the anterior temporal on both sides. Ventral tail of CIB 116089, CIB 116090 (for remaining part) and CIB 116091 immaculate instead of mottled with small dots. First to 10^th^ and the 12^th^ white crossbands on dorsum of CIB 116090 disconnected, forming moderate white dots covering two vertebral scales. Posterior chin shields suture of CIB 116090 barely exist. The dorsal bands are fewer in CIB 116091 (Fig. 12).

###### Cranial osteology.

The premaxilla of *B. suzhenae* sp. nov. is quite small and blunt, the ascending process of the premaxilla is well-developed, meeting the nasals at its dorsal edge. The nasal process of the premaxilla is not conspicuous. The nasal is peltate, with a blunt process on the lateral margin. The mesial process of the prefrontal is quite slender and pointed, narrowly reach the anterior tip of the frontal. Frontal triangular in shape from dorsal view. The distal process of the postorbital is slender and slightly anteriorly pointed, the basal part is in contact with the posterolateral marge of the frontal. A fenestra notch present on the posterolateral marge of the frontal. Two sides of the anterior surfaces of the parietal form the angle of approximately 120 degrees. The parietal is approximately “T” shaped; the lateral process is conspicuous and rectangular. The dorsal ridge of the parietal is more conspicuous in adults than in juveniles. The posterior end of the dorsal ridge merges at the mesial of the parietal in adults whereas separated in juveniles. The prefrontal surface of maxilla conspicuously upheaved. The supratemporal is flexed whereas the angular surface to the quadrate is obviously incrassated. The quadrate is quite short and stubby, the anterior angular surface to the supratemporal is extended. The ventral process of the basioccipital is trifurcate. The maxilla process (lateral process) of the palatine quite small whereas the choanal process is absent. The pterygoid is slender and medially curved, with the ectopterygoid process lost. The compound bone is quite stocky, the mesial crest and lateral crest are low and inconspicuous.

**Figure 13.**
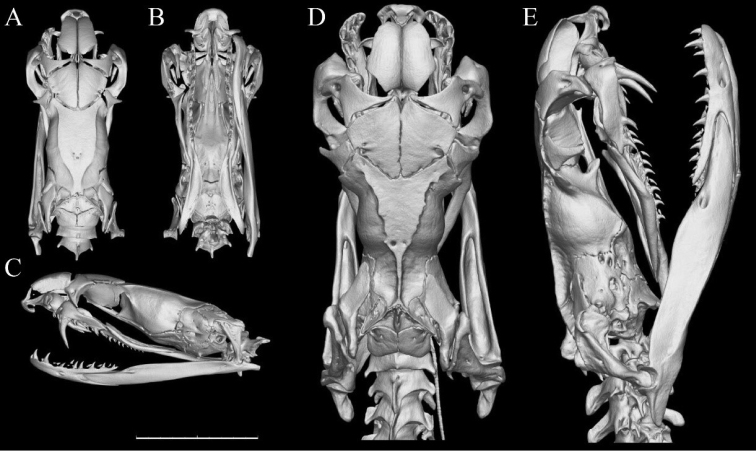
Three-dimensional reconstructed skull models of the holotype (**A–C**CIB 116088, subadult male) and paratype (**D, E**CIB 116090, adult female) of *Bungarus suzhenae* sp. nov. Dorsal (**A, D**), ventral (**B**), lateral (**C, E**) view. Scale bar: 10 mm.

The first fang is canaliculated and feebly curved behind. There are four or five replacement fangs posterior to the first. Three small solid teeth ranged on the posterior end of maxilla, decrease in size posteriorly and separated from the fang by a very large diastema. Palatine teeth 10 (11), pterygoid teeth 10 (9); dentary teeth 16 (15), 2, 3 and 4 largest, decrease in size posteriorly. (Fig. 13).

**Figure 14.**
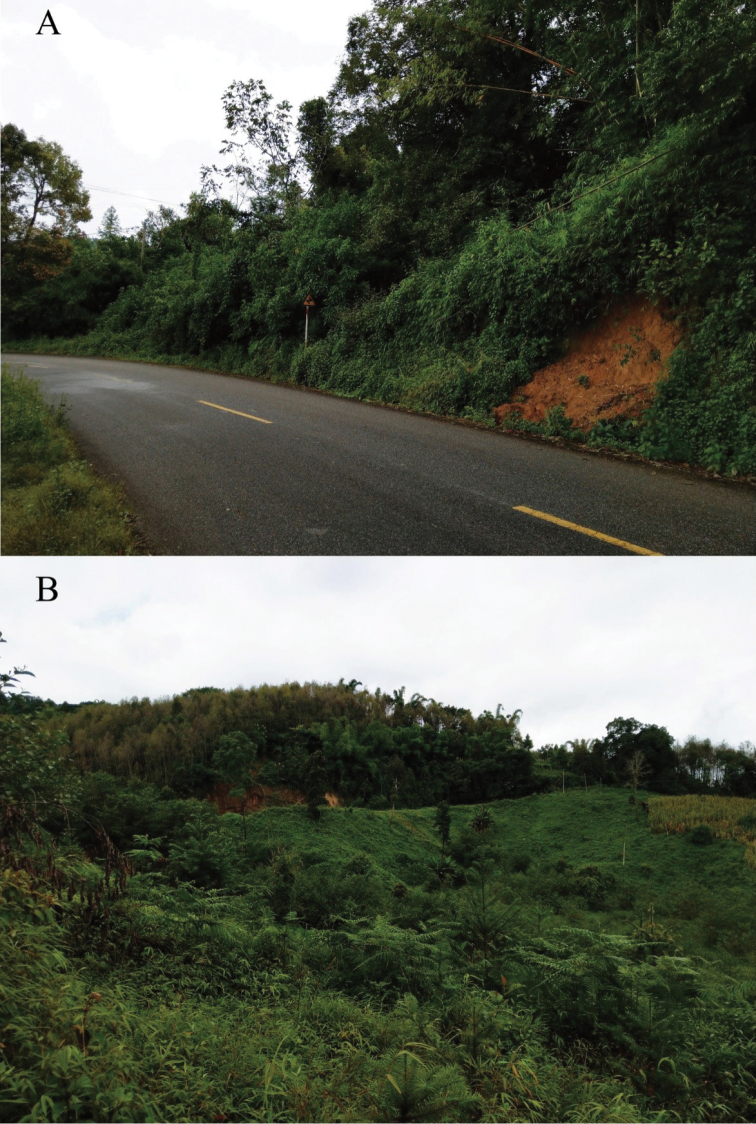
Habitats of *Bungarus suzhenae* sp. nov. Road **A** in Yingjiang County, Yunnan Province, China. Monsoon forest **B** in Yingjiang County, Yunnan Province, China.

The DOI numbers for ADMorph: 10.12112/R.0003 (CIB 116088, holotype) and 10.12112/R.0004 (CIB 116090, paratype).

###### Hemipenes.

Description is based on the adult male paratype CIB 116089 (Fig. 9J–L; SVL 1,140 mm). Hemipenis reaches 7^th^ subcaudal, slightly bilobed near top. Three zones of similar length of ornamentation exist: a distal calyculate zone, a spinose zone proximal to the sulcus bifurcation, and a basal zone. The line of demarcation between the calyculate zone and spinose zones is poorly defined. The calyculate zone is capitate; calyces well developed, gradually smaller and lesser keratinized towards the distal end; calyces nearest the sulcus intruding to the spinous zone by few ranks. The spinose zone is covered with large spines; large, elongated and pointed spines on hemipenes, in fang-shaped, gradually thinner from bases to tips; tips of large spine strongly keratinized, without distinct boundary with the main body of large spines; the spines adjacent to the calyces are nearly twice as large as the most proximal ones in a sulcate view. The hemipenis slightly constricts between the spinose zone and the basal zone. The basal zone is covered with numerous minute spines on the larger distal part, and smooth proximal region. The sulcus is forked distally along the spinose zone, with the bifurcation originating at a distance of about one large spine length; lips bearing calyces in calyculate zone, and small spines throughout the spinose region.

###### Etymology.

The specific epithet of the new species was named after Su-Zhen Bai, a famous powerful goddess of Chinese myth *The legend of the White snake* (白蛇传), in honor of her courage to true love and kindness to people. The common name is suggested as “Suzhen’s krait” in English and “素贞环蛇 (sù zhēn huán shé)” in Chinese.

###### Distribution and ecology.

*Bungarus suzhenae* sp. nov. was found in rice fields, streams in monsoon forest at elevation from 800 m to 1,560 m. This species is distributed in Yingjiang Country, Yunnan Province, China and Kachin State, Myanmar (Fig. 1). In captivity, they prey on eels like *Monopterus albus* and small snakes such *Xenochrophis flavipunctatus*, *Pareas* spp., but refuse mice and frogs.

## Discussion

Since the members of *Bungarus* are a group of deadly snakes, understanding their species diversity, species boundaries and geographic distribution is vital for saving human lives. Snakebites from kraits are known to have a high mortality, and the toxicology of their venom has been the subject of numerous publications (e.g., Mebs et al. 1971; Liu et al. 1998; Chang et al. 1999; Nirthanan et al. 2002; Jiang et al. 2011). An extreme and sad case for krait bites is that one individual of *Bungarus suzhenae* sp. nov. (CAS 221526) led to the death of famous herpetologist Joe Slowinski (Justin L. Lee 2020; personal communication). Many studies have indicated that the venom composition of *B. candidus* is different from *B. multicinctus* (e.g., Nirthanan et al. 2002; Tsai et al. 2002). Thus, a sound understanding of the species boundaries between these two species is necessary to provide the essential underpinnings for future research on their venom composition and antivenin development (Fry et al. 2003; Barlow et al. 2009; Williams et al. 2011; Casewell et al. 2013, 2014). However, identification of these *Bungarus* is still challenging. Our examination of the specimens from the *B. candidus*/*multicinctus*/*wanghaotingi* complex shows that the traditional characters used to diagnose the three taxa from each other (i.e., by the number of the white/black bands and the ventral scales) only work for *B. candidus* and *B. multicinctus*. Furthermore, the range of the white body bands of *B. wanghaotingi* overlaps with that of *B. candidus* and *B. multicinctus* (Table 1) and may be unreliable for identification without further examination of other characters. As for *B. suzhenae* sp. nov., the range of the number of crossbands also overlaps with that of the *B. candidus*/*multicinctus*/*wanghaotingi* complex except *B. candidus* (Table 1).

Thorough morphological examination and comparisons are essential for taxonomy of *B. candidus*/*multicinctus*/*wanghaotingi* complex. The topology of molecular phylogeny in Xie et al. (2018) is similar to the topology in this study. The samples from Java, Indonesia, Thailand, and Vietnam form a lineage (*B. candidus* lineage) sister to the lineage (*B. wanghaotingi* lineage) including samples from southwestern and southern China, Laos, Thailand and Vietnam. However, Xie et al. (2018) concluded that the latter lineage is *B. candidus* without morphological comparisons with typical *B. candidus* from Indonesia and Peninsular Malaysia. Thus, the distribution report of “*B. candidus*” in the southwestern and southern China mentioned by Xie et al. (2018) should be revised into *B. wanghaotingi*. And similarly, the records of “*B. multicinctus*” in Vietnam, Laos, and Thailand (Uetz and Hošek 2017) are supposed to be *B. wanghaotingi*.

The “*B. candidus*” from Southern and Central Vietnam mentioned by Nguyen et al. (2017) are paraphyletic based on COI sequences data when *B. candidus* from Peninsular Malaysia (SYNU R180411, which is monophyletic with Javanese *B. candidus* in cyt *b* phylogeny) is included. Most specimens (e.g., ITBCZ 900) are monophyletic with typical *B. wanghaotingi* from China, Laos and Southern Thailand. But one specimen (KIZ 100) is monophyletic with two *B. candidus* from Peninsular Malaysian and Cambodia. This suggests a large sympatric zone from Southern Thailand to Southern Vietnam where *B. wanghaotingi* and *B. candidus* are morphologically similar in having wide crossbands but genetically paraphyletic (Fig. 1; Nguyen et al. 2017). However, the hemipenial morphology of wide crossbanded *B. wanghaotingi* ITBCZ 900 (fig. 3 in Nguyen et al. 2017) is quite similar with typical *B. wanghaotingi* by large spines on hemipenes relatively short, and weakly keratinized. Southern Indochina populations of *B. wanghaotingi* have wider crossbands on body than typical *B. wanghaotingi* in the north and this is possibly due to different selective constraints from habitats at lower latitude. This is similar to the discussion on *B. candidus* and *B. javanicus* by Kuch and Mebs (2007). And similar scenario for that northern *Bungarus* species *B. suzhenae* and *B. multicinctus* which have narrower body bands. The difference between hemipenial morphology of *B. candidus* (RH06153) and *B. wanghaotingi* (both typical and Southern Indochina populations) further confirmed the identification of RH06153 when sequence data was unavailable.

Based on comprehensive morphological comparisons of specimens and molecular evidence, we identified additional morphological characters that can be used to be help identify these taxa (e.g., dental morphology, the patterns on head, ventral body and tail, and large spines on hemipenis). We note that the number of maxillary teeth is taxonomically significant in identifying species of *Bungarus* and might indicate divergences in feeding and/or defense behavior. *B. suzhenae* sp. nov. only possesses three posterior maxilla teeth, a character state shared by *B. fasciatus* and *Ophiophagus hannah*. However, all members of the *B. candidus*/*multicinctus*/*wanghaotingi* complex possess four posterior maxilla teeth, a character state that is likely a synapomorphy in comparison to *B. suzhenae* sp. nov. (Fig. 8).

The species of the *B. candidus*/*multicinctus*/*wanghaotingi* complex show feeble genetic divergences (Suppl. material 1: Table S3), with the uncorrected pairwise distances of cyt *b* between *B. candidus*, *B. multicinctus* and *B. wanghaotingi* ranging from 1.6%–3.3%. These distances are lower than those between other *Bungarus* species (ranging from 9.7% to 28.6%). However, morphological comparisons showed clear differences between these closely related species. According to Kuch (2007), *B. candidus*, *B. multicinctus* and *B. wanghaotingi* diverged between Middle to Late Pleistocene (> 0.8–1.5 MYA). During the Pleistocene, southeast Asia and southern China went through very frequent sea level oscillations (Voris 2000; Inger and Voris 2001) and the *B. candidus*/*multicinctus*/*wanghaotingi* complex may have experienced several hybridization events, introgression or incomplete lineage sorting. We encourage future studies to examine the *B. candidus*/*multicinctus*/*wanghaotingi* complex using genomic data, ecological niche modelling and broader sampling, which would help to better understand the evolutionary history of these medically important snakes.

For easier identification, an updated Key to Kraits based on Slowinski (1994) was compiled (note that the identification of this key is still experimental, confirmation by comparing more characters or by sequencing is strongly recommended):

**Table:** 

la	More than 13 dorsal scale rows	**2**
lb	13 dorsal scale rows	***Bungarus flaviceps***
2a	17 or 19 dorsal scale rows	**3**
2b	15 dorsal scale rows	**5**
3a	Loreal plate present	***B. persicus***
3b	Loreal plate absent	**4**
4a	White bands on body	***B. sindanus***
4b	White spots on body	***B. walli***
5a	Subcaudals divided	**6**
5b	Subcaudals single	**7**
6a	Inversely V-shaped light on dorsal head	***B. slowinskii***
6b	Without inversely V-shaped light on dorsal head	***B. bungaroides***
7a	Vertebral row of dorsal scales enlarged anteriorly	**8**
7b	Vertebral row of dorsal scales not enlarged anteriorly	***B. lividus***
8a	Bands or rings on body	**9**
8b	No bands or rings on body	***B. niger***
9a	Light bands or rings white	**10**
9b	Light bands yellow	***B. fasciatus***
10a	Rings on body	***B. ceylonicus***
10b	Bands on body	**11**
11a	More than 20 white bands	**12**
11b	11–14 white bands	***B. magnimaculatus***
12a	More than 26 white bands	**13**
12b	Less than 26 white bands	***B. candidus* , or wide white crossbanded *B. wanghaotingi* from Southern Indochina and Peninsular Malaysia**
13a	At least some white bands occurring in pairs	***B. caeruleus***
13b	White bands not in pairs	**14**
14a	White bands equidistant from each other along body	***B. andamanensis***
14b	White bands closer to each other posteriorly than anteriorly	**15**
15a	Three posterior maxilla teeth	***B. suzhenae***
15b	Four posterior maxilla teeth	**16**
16a	Ventral surface of tail immaculate white or with dots, large spines on hemipenes mostly pointy	***B. wanghaotingi* (typical populations from Northern Indochina and China)**
16b	Ventral surface of tail with broad dark bands or patches, large spines on hemipenes papilla-like in shape and blunt	***B. multicinctus***

## Supplementary Material

XML Treatment for Bungarus candidus

XML Treatment for Bungarus multicinctus

XML Treatment for Bungarus wanghaotingi

XML Treatment for Bungarus suzhenae

## Appendix 1

Specimens examined for measurements and morphology; localities as originally stated

***B. caeruleus*.** (9 specimens). “Indes Orientalis” MNHN 7688. “Bengale” MNHN 3952, MNHN 7687. “Malabar” MNHN 7687. “Pondicheri” MNHN 7686. “Pakistan occ” MNHN 1962.239, MNHN 1962.236, MNHN 1962.238, MNHN 1962.237. “Birma” MNHN1893-413.

***B. ceylonicus*.** (1 specimens). “Sri Lanka” MNHN 4259 (1872-32).

***B. fasciatus*.** (1 specimens). “Bengkalis, Siak, Indonesia” RMNH 1667.

***B. candidus*.** (20 specimens). “Palembang, Sumatra, Indonesia” RMNH 11416. “Java, Indonesia” NMW 27730:2, NMW 27730:7, NMW 27730:3, NMW 27730:4; NMW 27740:5, NMW 27740:4, NMW 9486:4, NMW 9486:3, NMW 27730:5, NMW 27730:1, NMW 9486:1, NMW 9486:6, NMW 9486:5, NMW 9486:2, NMW 27711:1, NMW 27711:2, NMW 27711:3. “Johor, Malaysia” SYNU R180411. “Quang Binh, Vietnam” RH06153.

***B. multicinctus*.** China: (24 specimens). “Anhui, China” CIB 12209; “Chongqing, China” CIB 12215. “Fujian, China” CIB 12212, CIB 12204, CIB 12203, CIB 12207, CIB 12206, CIB 12205, CIB DL18090209, CIB DL18090210; “Guangdong, China” CIB 12191, CIB 12194; “Guangxi, China” CIB 12192, CIB 93924, CIB 93923, CIB 104228; “Zhejiang, China” CIB 12208; “Jiangxi, China” CIB 12210, CIB 12211; “Hainan, China” CIB 12197, SYNU R180305; “Guizhou, China” CIB 83793; “Hunan, China” CIB 12213, CIB 12214.

***B. wanghaotingi*.** China: (16 specimens). “Guangxi, China” CIB FCDZ20170806, CIB 104227. “Yunnan, China” CIB 12216, CIB 12201, CIB ML20170801, CIB MLMY20170801, CIB JCR36, CIB JCR36-2, CIB DL2019051401, CIB DL20190525, CIB DL2019070301, CIB DL20190522, CIB JCR2019062003, CIB JCR2019062311, CIB JCR2019061703, CIB JCR2019061807.

***B. suzhenae* sp. nov. China**. (4 specimens). “Yingjiang, Yunnan, China” CIB 116088–CIB 116091.

Specimens checked hemipenes:

***B. multicinctus*.** “Guangdong, China” CIB 12191. “Shangyou, Jiangxi, China” CIB 12211. “Nanning, Guangxi, China” CIB 104228. “Lishui, Zhejiang, China” CIB DL2019051701.

***B. wanghaotingi*. China.** “Luodian, Guizhou, China” CIB 83793. “Beiliu, Guangxi, China” CIB 104227. “Mengla, Yunnan, China” CIB MLML20170801.

***B. suzhenae* sp. nov.** “Yingjiang, Yunnan, China” CIB 116089.

***B. candidus*** “Phong Nha-Ke Bang National Park Administration, Quang Binh Province, Vietnam” RH06153.

## Supplementary materials

Supplementary material 1 Tables S1–S4 Data type phylogenetic data Explanation note **Table S1.** DNA sequences used in this study. **Table S2.** Best evolution models of each partition combination. **Table S3.** Uncorrected p-distances between Bungarus species based on 1069 base pairs from the mitochondrial genes cyt b. **Table S4.** Uncorrected p-distances between Bungarus species based on 613 base pairs from the mitochondrial genes COI. https://binary.pensoft.net/file/521383This dataset is made available under the Open Database License (http://opendatacommons.org/licenses/odbl/1.0/). The Open Database License (ODbL) is a license agreement intended to allow users to freely share, modify, and use this Dataset while maintaining this same freedom for others, provided that the original source and author(s) are credited.Ze-Ning Chen, Sheng-Chao Shi, Gernot Vogel, Li Ding, Jing-Song Shi
